# Molecular analysis of the emergence of pandemic *Vibrio parahaemolyticus*

**DOI:** 10.1186/1471-2180-8-110

**Published:** 2008-06-30

**Authors:** E Fidelma Boyd, Ana Luisa V Cohen, Lynn M Naughton, David W Ussery, Tim T Binnewies, O Colin Stine, Michelle A Parent

**Affiliations:** 1 Department of Biological Sciences, University of Delaware, Newark, DE 19716, USA; 2 Department of Microbiology, UCC, National University of Ireland-Cork, Ireland; 3 Center for Biological Sequence Analysis, BioCentrum, Technical University of Denmark, DK-2800 Kgs, Lyngby, Denmark; 4 Department of Epidemiology and Preventive Medicine, University of Maryland School of Medicine, Baltimore, MD 21201, USA; 5 Department of Medical Technology, University of Delaware, Newark, DE 19716, USA

## Abstract

**Background:**

*Vibrio parahaemolyticus *is abundant in the aquatic environment particularly in warmer waters and is the leading cause of seafood borne gastroenteritis worldwide. Prior to 1995, numerous *V. parahaemolyticus *serogroups were associated with disease, however, in that year an O3:K6 serogroup emerged in Southeast Asia causing large outbreaks and rapid hospitalizations. This new highly virulent strain is now globally disseminated.

**Results:**

We performed a four-way BLAST analysis on the genome sequence of *V. parahaemolyticus *RIMD2210633, an O3:K6 isolate from Japan recovered in 1996, versus the genomes of four published *Vibrio *species and constructed genome BLAST atlases. We identified 24 regions, gaps in the genome atlas, of greater than 10 kb that were unique to RIMD2210633. These 24 regions included an integron, f237 phage, 2 type III secretion systems (T3SS), a type VI secretion system (T6SS) and 7 *Vibrio parahaemolyticus *genomic islands (VPaI-1 to VPaI-7). Comparative genomic analysis of our fifth genome, *V. parahaemolyticus *AQ3810, an O3:K6 isolate recovered in 1983, identified four regions unique to each *V. parahaemolyticus *strain. Interestingly, AQ3810 did not encode 8 of the 24 regions unique to RMID, including a T6SS, which suggests an additional virulence mechanism in RIMD2210633. The distribution of only the VPaI regions was highly variable among a collection of 42 isolates and phylogenetic analysis of these isolates show that these regions are confined to a pathogenic clade.

**Conclusion:**

Our data show that there is considerable genomic flux in this species and that the new highly virulent clone arose from an O3:K6 isolate that acquired at least seven novel regions, which included both a T3SS and a T6SS.

## Background

*Vibrio parahaemolyticus *is a Gram-negative halophilic, aerobic bacterium that is distributed in marine and estuarine environments worldwide [1]. In the 1950s, Fujino demonstrated that *V. parahaemolyticus *was the etiological agent responsible for a gastroenteritis outbreak in Osaka, Japan. Presently, in Taiwan, Japan and other South East Asian countries, *V. parahaemolyticus *cause over half of all food poisoning outbreaks of bacterial origin [2,3]. Baross and Liston in the late 1960s identified *V. parahaemolyticus *in seawater, sediments and shellfish in the United States [4,5]. Today, *V. parahaemolyticus *is the leading cause of seafood-associated bacterial gastroenteritis in the United States. *V. parahaemolyticus *can also cause serious wound infections resulting in necrotizing fasciitis when wounds are exposed to *V. parahaemolyticus *contaminated water [6-8]. Although less common, *V. parahaemolyticus *can cause fatal septicemia in immune compromised hosts [6,7]. Most isolates of *V. parahaemolyticus *are non-pathogenic and only a small number can cause infections in humans [1]. Clinical isolates of *V. parahaemolyticus *produce beta type hemolysis on blood agar (Wagatsuma agar) called the Kanagawa-phenomenon (KP), which is linked to the production of a thermostable direct hemolysin (TDH) [9-11]. TDH damages eukaryotic cells by acting as a pore forming toxin that alters the ion balance of cells [12]. The presence of the *tdh *gene, which encodes TDH is often used as a diagnostic tool to identify pathogenic isolates of *V. parahaemolyticus*. Five sequence variants of *tdh *(named *tdh1 *to *tdh5*) have been identified, however only *tdh2 *appears to have a high-level of transcription [13,14]. In the 1980s, several cases of gastroenteritis caused by hemolytic KP-negative TDH-negative *V. parahaemolyticus *isolates were reported [11]. These isolates contained a TDH-related hemolysin (TRH) encoded by *trh*, which showed 69% sequence similarity with *tdh *[11]. TDH and TRH are considered the main virulence factors of *V. parahaemolyticus *and strains can contain either TDH or TRH or both [15-19]. Although isolates that do not contain *tdh *or those that have a deletion in *tdh *are still cytotoxic to cells. Hence, the overall mechanism involved in the organism's pathogenesis remains unclear.

Analysis of the complete genome sequence of *V. parahaemolyticus *RIMD2210633, a clinical isolate recovered in Japan in 1996, identified a type III secretion system (T3SS) on each chromosome designated T3SS-1 and T3SS-2 [20]. Subsequently, the functional significance of both T3SSs was determined using deletion mutants [21]. The T3SS-1 deletion mutants had significantly decreased cytotoxic activity compared with that of the wild type [21]. The T3SS-2 deletion mutants showed diminished intestinal fluid accumulation, in an enterotoxicity assay using the rabbit ileal loop test, whereas T3SS-1 mutants were similar to the wild type [21]. In addition, a number of effector proteins for these T3SSs have been identified [22-24]. T3SS-1 is present in both clinical and environmental isolates and has a percent G+C content similar to the rest of the genome indicating that this region is ancestral to the species [20]. Henke and Bassler [25] found that unlike other T3SSs in pathogenic *E. coli*, which are activated by quorum sensing, T3SS-1 in *V. parahaemolyticus *is repressed at high cell densities.

Associated with T3SS-2 encoded on chromosome 2 are Tdh1 and Tdh2, as well as a cytotoxic necrotizing factor, an exoenzyme T, and at least five transposases [20]. The presence of transposases and a G+C content of 40% (less than the overall genome), suggests that T3SS-2 may be a integrative element similar to pathogenicity islands identified in pathogenic *E. coli, S. enterica*, and *V. cholerae*, which we named *Vibrio parahaemolyticus *island-7 (VPaI-7) [20,26]. T3SS-2 is present predominantly in the *V. parahaemolyticus *O3:K6 highly virulent strains recovered after 1995, whereas most clinical isolates recovered before 1995 do not encode T3SS-2 indicating that the region is not essential for virulence, but may enhance virulence when present [20].

Serotyping of *V. parahaemolyticus *isolates has identified more than 13 O antigen groups and 71 K antigen types [27]. Up until 1995, *V. parahaemolyticus *associated gastroenteritis was caused by many different serogroups, although in some geographic regions specific serogroups predominated. For example, in the United States a predominance of the O4 serogroup among clinical isolates was apparent [28-32]. In 1995, an outbreak of *V. parahaemolyticus *infections occurred in Calcutta, India, which caused rapid hospitalization of those infected and were caused by a single serotype, a new O3:K6 highly virulent strain [33]. Since 1995, a global dissemination of this *V. parahaemolyticus *new highly virulent strain is evident since it has now been isolated throughout Asia, America, Africa, and Europe [3,29,34-40]. For example, in 1998, the new highly virulent strain was responsible for a large outbreak of gastroenteritis in Galveston Bay, Texas [29]. Later on that year, the highly virulent strain was responsible for large gastroenteritis outbreaks in Long Island Sound-Connecticut, New York, and New Jersey [41]. In 2005, the highly virulent strain caused a major outbreak in Chile with over 1,000 cases [3]. Non-O3:K6 pathogenic isolates recovered since 1995, including O4:K68, O1:KUT, and O1:K25 serotypes, have been shown to be closely related to the new highly virulent O3:K6 strain based on molecular typing schemes and phylogenetic approaches [29,30,37-39,42-44].

Previously, it was thought that *V. parahaemolyticus *was confined to tropical climates, however recent studies report the recovery of O3:K6 isolates from the water in Southern Chile and Alaska, that up until now were considered too cold to support the growth of this organism [35,45,46]. These recent discoveries suggest a change in the organism's ability to adapt and survive in colder environments. Indeed the ability of *V. parahaemolyticus *to survive and proliferates in its environmental niches, in shellfish and in the human intestine may have resulted from the acquisition of regions encoding novel traits which are differentially regulated in different niches. Additionally, the spread of the organism is another indication of global warming, which is likely to play a role in increasing *V. parahaemolyticus *distribution and occurrence.

First, we used a two step genomic approach to elucidate the genomic changes that may have resulted in the emergence of the new highly virulent O3:K6 and related strains. We performed *in silico *whole genome comparisons of *V. parahaemolyticus *RIMD2210633 versus the genome sequences of *V. cholerae *N16961, *V. vulnificus *YJ016 and CMCP6, and *V. fischeri *ES114. We constructed genome BLAST atlases of each species to determine regions unique to *V. parahaemolyticus*. We uncovered 24 regions greater than 10 kb that were unique to RIMD2210633 and absent from the other *Vibrio *species examined. These included functionally distinct regions such as the class 1 integron, f237-like phages, *Vibrio parahaemolyticus *genomic island regions (VPaI-1 to VPaI-7), a lipopolysaccaride (LPS)/capsule polysaccharide (CPS) region, two osmotic stress response clusters, two T3SSs and a T6SS. Next, we compared the RIMD2210633 genome sequence to that of AQ3810, an O3:K6 strain isolated in 1983, to elucidate the steps involved in the emergence of the globally disseminated O3:K6 highly virulent strain. This analysis identified several regions unique to one isolate or the other. Molecular analysis of the distribution of regions unique to RIMD2210633 among 42 natural isolates revealed that only regions encoding integrase or transposase genes (7 island regions) were variably present. We reconstructed the phylogeny of the 42 isolates based on multilocus sequence analysis, and mapped the distribution of the 7 island regions, which showed that these regions were acquired by the new O3:K6 highly virulent strain and predominant in one clade.

## Results and Discussion

### Comparative genome analysis of *V. parahaemolyticus *RIMD2210633 versus *V. cholerae *N16961, V. vulnificus YJ016 and CMCP6, and *V. fischeri *ES114

**Figure 1:**
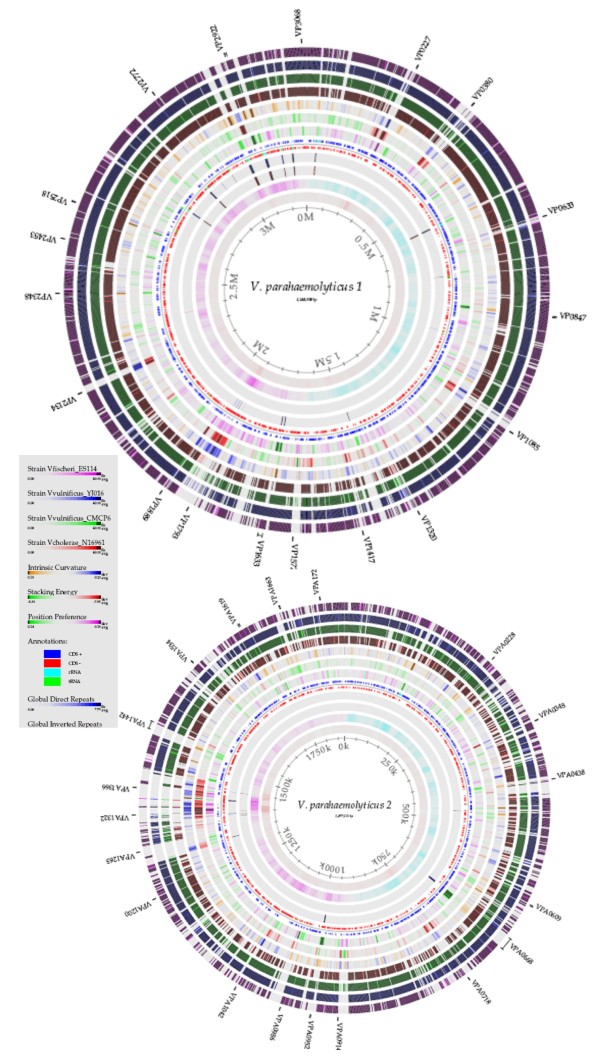
**Genome BLAST Atlas of *V. parahaemolyticus *RIMD2210633 as reference strain (inner most circle) versus *V. fischeri *ES114 (outer most circle purple), *V. vulnificus *strains YJ016 and CMCP6 (2nd navy and 3rd green circles) and *V. cholerae *N16961 (4th circle brown) for chromosome 1 (a) and chromosome 2 (b)**[**60**]**.** The gaps or holes in the outer four circles represent regions present in *V. parahaemolyticus *strain RIMD2210633 that are absent from the other three species. The innermost circles show DNA structure features, DNA intrinsic curvature (circle 5), DNA stacking energy (circle 6), DNA position preference (circle 7), positive and negative coding strands are indicated by dark blue and red circle. Global direct and global inverted repeats are represented by circles 9 and 10, respectively and the two inner most circles represent GC shew and AT content, respectively.

Systematic BLAST analysis was carried out for each of the ORFs of *V. parahaemolyticus *RIMD2210633 compared with each of the ORFs from the genome sequences of *V. cholerae *N16961, *V. vulnificus *YJ016 and CMCP6, and *V. fischeri *ES114. This four-way BLAST analysis was used to construct genome BLAST atlases of chromosome 1 and 2 of the four *Vibrio *species with *V. parahaemolyticus *RIMD2210633 as a reference (Fig. 1). The four outer circles of solid color represent conserved proteins of the BLASTed genomes for both chromosome 1 and 2. The outer most circle represents the *V. fischeri *ES114 genome (purple circle), the next two circles represents *V. vulnificus *YJ016 and CMCP6 (navy and green circles), followed by the fourth circle (brown circle), which represents *V. cholerae *N16961. The innermost circles show DNA structure features, repeat sequences and base composition properties of the reference *V. parahaemolyticus *RIMD2210633 (Fig. 1). It is of interest to note that chromosome 1 shows a higher level of overall conservation among the species examined than chromosome 2 indicating that a lot of species specific genes lie on chromosome 2. There are approximately 44 gap regions (greater than 1 kb) on chromosome 1 and 29 gap regions (greater than 1 kb) on chromosome 2, common to all four outer circles and these gaps represent regions of the *V. parahaemolyticus *RIMD2210633 chromosomes that are unique being absent from all other isolates examined. Differences in these regions in their DNA structural features such as intrinsic curvature, stacking energy and position preference correlate with some of the gap regions and represent phages, integrons and genomic islands, that is signatures of foreign DNA acquired by horizontal transfer (Fig. 1).

Of the 73 gap regions, our analysis uncovered 24 regions greater than 10 kb that are present in RIMD2210633 and absent from the other species examined, that is the gap regions in the four outer circles in Figure 1 (Table 1). Of the 24 regions identified, 11 regions encoded an integrase or transposase, 9 regions had aberrant GC content (45 ± 3%), 7 regions of which had lower G+C content compared to the rest of the genome suggesting that these regions were acquired by horizontal gene transfer (Table 1). The 24 regions included 14 previously identified: lipopolysaccharide and capsule polysaccharide gene clusters, a class 1 integron, 2 f237 phage regions, 2 osmotic stress response gene clusters, 2 T3SSs, and the *Vibrio parahaemolyticus *island (VPaI) regions (Table 1) [20,26,47]. The 10 additional regions unique to *V. parahaemolyticus *RIMD2210633 included 2 regions encoded on chromosome 1 and 8 regions encoded on chromosome 2 (Table 1). On chromosome 1, region VP0081 to VP0092 encodes mainly hypothetical proteins of unknown function; VP0081 encodes a homologue of a hyper osmotic shock protection protein. Region VP1386 to VP1420 encodes hemo utilization/adhesion proteins, OmpA, a ClpA/B type protease, a BfdA homologue, a putative IcmF-related protein and related type VI secretion system (T6SS) proteins (VP1401 to VP1409), which is predicted to be involved in intracellular trafficking, secretion, and vesicular transport in other Gram-negative pathogens. On chromosome 2, region VPA0434 to VPA0458 encodes a large number of homologues of genes involved in degradation processes. Region VPA887 to VPA0914 encode proteins that show homology to phage f237 on chromosome 1. Within region VPA0950 to VPA0962 are homologues of biofilm associated proteins among others. Region VPA0989 to VPA0999 contains homologues of a number of peptidase, lipase and amylase genes, and region VPA1440 to VPA1444 encode a type I secretion system (Table 1). Region VPA1503 to VPA1513 contained a type 1 pilin homologue similar to Pap pilin identified in *Burkholderia pseudomallie, Pseudomonas spp *and *Yersinia *spp. Region VPA1559 to VPA1583 encodes a number of proteins with a possible role in antibiotic resistance and region VPA1652 to VPA1679 contains a ferric uptake system.

**Table 1:** Chromosomal regions unique to *V. parahaemolyticus *strains RIMD2210633 and AQ3810

**ORFs**	**Region type**	**Size**	**% GC**	**Int**	**AQ3810**	**Reference**
**Chromosome I**						

VP0081 – VP0092	NK	10	48	-	Present	This study
VP0218 – VP0234	LPS	46	40	-	Present	This study
VP0380 – VP0403	VPaI-1	24	42	Int	Absent	[26]
VP0634 – VP0643	VPaI-2	10	45	Int	Absent	[26]
VP1071 – VP1095	VPaI-3	32	42	Int	Partial	[26]
VP1386 – VP1420	T6SS	57	43	-	Absent	This study
VP1549 – VP1590	phage f237	25	46	Int	Absent	[33]
VP1658 – VP1702	T3SS-1	35	47	-	Present	[26]
VP1719 – VP1728	Osmotolerance	12	46	-	Present	[47]
VP1787 – VP1865	Integron class-1	48	40	Int	Absent	This study
VP2131 – VP2144	VPaI-4	17	39	Int	Absent	[26]
VP2900 – VP2910	VPaI-5	12	38	Int	Absent	[26]

**Chromosome II**						

VPA0434 – VPA0458	Degradative	29	46	Int	Partial	This study
VPA0887 – VPA0914	phage f237-like	16	47	Int	Present	This study
VPA0950 – VPA0962	Biofilm	22	47		Present	This study
VPA0989 – VPA0999	Gametolysin	18	45		Present	This study
VPA1102 – VPA1115	Osmotolerance	17	46		Present	[47]
VPA1253 – VPA1270	VPaI-6	27	43	Int	Absent	[26]
VPA1312 – VPA1395	VPaI-7 (T3SS-2)	81	39	Tnp	Present	[26]
VPA1403 – VPA1412	CPS	13	47		Present	This study
VPA1440 – VPA1444	Type I secretion	20	47		Present	This study
VPA1503 – VPA1521	Type I pilus	20	45		Present	This study
VPA1559 – VPA1583	Multidrug efflux	22	46		Present	This study
VPA1652 – VPA1679	Ferric uptake	25	50		Present	This study

We also constructed genome BLAST atlases of all 28 genomes available for members of the family *Vibrionaceae *in the database, this included eight additional species of the *Vibrionaceae *family. *V. parahaemolyticus *RIMD2210633 as reference strain (inner most circle) versus *V. parahaemolyticus *AQ3810, *V. cholerae *1587, AM-19226, MAK757, MO10, MZO-2, MZO-3, B33, NCTC8457, RC385, O395, V51, V52, 623–39, and 2740–80, *V. harveyi *ATCC BAA116, *V. alginolyticus *12G01, *Vibrio *sp. Ex25, *V. vulnificus *CMCP6 and YJ016, *V. splendidus *12B01, *Vibrio *sp. MED222, *V. fischeri *ES114, *V. salmonicida *LF1238, *V. angustum *S14, *P. profundum *SS9 and 3TCK for chromosome 1 (a) and chromosome 2 (b), in which each gap region can be zoomed in on to examine in detail (see Additional file 1 &2) [48].

Most of the 24 gap regions remained unique to *V. parahaemolyticus*, exceptions were noted (see Additional file 1 and 2). For example, on chromosome 1 region VP0081 to VP0092 is present in *V. alginolyticus *12G01 and *Vibrio *sp. Ex25, the osmotolerance gene clusters are partially present in *V. alginolyticus *12G01, *V. harveyi *ATCC BAA116 and *Vibrio *sp. Ex25, and the T3SS-1 and T6SS (VP1388 to VP1414) are present in *V. alginolyticus, V. harveyi *and *Vibrio *sp. Ex25 (see Additional file 1 and 2). On chromosome 2, region VPA0950 to VPA0962 was present in *Vibrio *sp. Ex25, homology to VPaI-7 within the T3SS-2 region (VPA1332 to VPA1355 and VPA1358 to VPA1370) is present in *V. cholerae *strains 1587, AM-19226, V51 and 623–39. Region VPA1440 to VPA1442 is present in *Vibrio *sp. Ex25. Region VPA1503 to VPA1521, which encodes a type I pilin is present in *V. alginolyticus, V. harveyi *and *Vibrio *sp. Ex25 (see Additional file 1 and 2).

### Genome sequence of *V. parahaemolyticus *AQ3810

The previously published *V. parahaemolyticus *genome sequence is from RIMD2210633 a *tdh *positive, *trh *and urease negative O3:K6 clinical isolate of the highly virulent clone recovered in Japan in 1996. In order to unravel events at the genome level that may have lead to the emergence and dissemination of the new highly virulent strain, we sequenced the genome of AQ3810, a *tdh *positive, *trh *and urease negative O3:K6 isolate recovered in Japan in 1983 for comparison. The complete genome sequence of AQ3810 is 5.8 Mb and 5509 proteins have been annotated within its genome compared with the 5.2 Mb genome of RIMD2210633, which has 4832 annotated proteins. There is extensive sequence homology between the two sequences, however genomic differences were noted. As had been found within other *Vibrio *species, the gene capture system, the -integron encoded in RIMD2210633 and AQ3810 do not share any significant sequence similarity.

We examined the genome of AQ3810 for the presence of the 24 regions identified as unique to RIMD2210633 from our species comparisons (Table 1). Of the 24 regions, 8 regions were absent from AQ3810, 5 genomic islands (VPa-1, VPaI-2, VPaI-4, VPaI-5 and VPaI-6), ORFs VP1386 to VP1420, which encodes T6SS, the class 1 integron, and phage f237 encoded on chromosome 1 (Table 1). These data confirm our previous result that VPaI-1, VPaI-4, VPaI-5 and VPaI-6 are unique to the new highly virulent strain recovered after 1995 [26]. For example, two of the missing regions, VPaI-1 and VPaI-4, integrate at a tRNA-met (VP0404.1) and tRNA-ser locus (VP2130.1), respectively in RIMD2210633, however, in AQ3810, both of these tRNA sites are empty (see Additional file 3). The other two missing regions VPaI-5 and VPaI-6 regions are located between core chromosomal ORFs VP2889 and VP2911, and VPA1252 and VPA1271, respectively in RIMD2210633, while in AQ3810, the homologues of these genes are contiguous indicating that these sites are empty (see Additional file 3).

Two VPaIs were rearranged. One, the VPaI-2 region (VP0634 to VP0643), is present at the tmRNA gene (*ssrA*) in RIMD2210633, a gene that encodes both tRNA and mRNA properties. In AQ3810, at this same locus, the first three genes of this region are present (VP0634 to VP0636), which encode homologues of a nitrilase/cyanide hydratase, OmpA and LysR, but the remaining genes are replaced by a novel region encoding an integrase (see Additional file 4). A second island region, VPaI-3 (VP1071 to VP1095) present at a second tRNA-ser locus (VP1070.1) in both RIMD2210633 and AQ3810 has 21 genes in common (ORFs VP1074 to VP1095) and 6 genes, two novel integrases and four hypothetical proteins, are adjacent to the tRNA-ser locus in AQ3810 (see Additional file 4).

Two additional regions named VPaI-8 and VPaI-9 were identified in AQ3810 (see Additional file 4). VPaI-8 is a 17 kb region located between homologues of VP3057 and VP3058 and contains ORFs A79_5175 to A79_5191, which encode a number of hypothetical proteins, homologues of SMF and KAP proteins, and two integrases separated by a single ORF (see Additional file 4). VPaI-9 is a 22 kb region integrated between homologues of VP0006 and VP0007. VPaI-9 encodes an integrase, an excisionase, a helicase and a type I restriction modification system.

ORFs VP1386 to VP1420 are absent from AQ3810. This regions encodes T6SS (ORFs VP1401 to VP1409) and a range of proteins that could be translocated by this system; hemo utilization/adhesion proteins, OmpA, a ClpA/B type protease, a BfdA homologue, a putative IcmF-related protein. This suggests the presence of an additional virulence mechanism in the highly virulent O3:K6 clone. T6SSs have been identified in a range of Gram-negative pathogens including pathogenic *V. cholerae *and in that species T6SS translocates a bacterial host protein into host cells that cross link actin [49].

### Distribution of VPaI-2, VPaI-3 and VPaI-7

**Figure 2:**
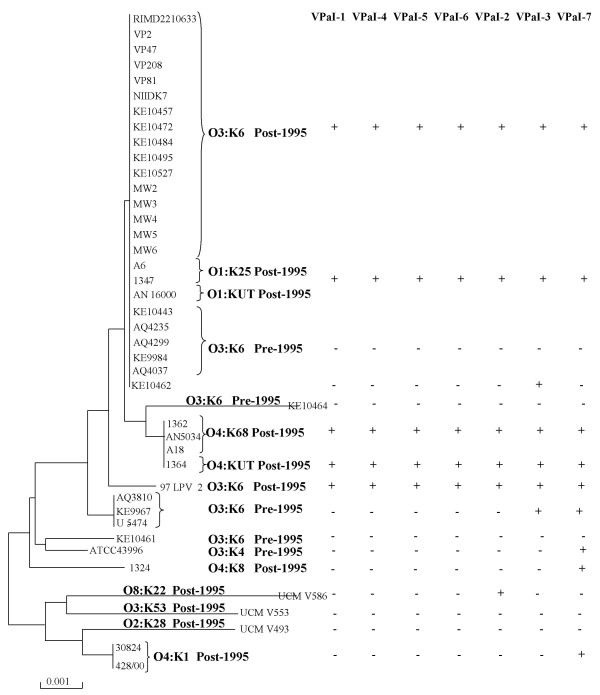
**Evolutionary relationships of *V. parahaemolyticus *isolates based on the concatenated housekeeping gene tree.** Phylogenetic trees were constructed using the neighbor-joining method based on the based on Kimura 2-parameter distance using MEGA-3. The plus and minus signs represent the presence and absence of VPaIs among our collection of isolates.

Previously, we examined the distribution of VPaI-1, VPaI-4, VPaI-5 and VPaI-6 among a worldwide collection of *V. parahaemolyticus *isolates and found that these regions are unique to 24 isolates of the highly virulent O3:K6 clone [26]. We determined the distribution of VPaI-2, VPaI-3 and VPaI-7 using primer pairs described in Table 2. Of the 42 *V. parahaemolyticus *isolates examined by PCR assays using two primer pairs encompassingVPaI-2, 27 isolates gave positive PCR bands. These isolates were recovered post-1995 and include the 24 isolates that were previously shown to contain VPaI-1, VPaI-4 to -6 (Fig. 2). VPaI-2 was also present in isolates UCMV586 and 1324, O8:K22 and O4:K6 isolates recovered after 1995, and ATCC43996, an O3:K4 clinical isolate from recovered in the UK in 1970 (Fig. 2). The presence of VPaI-2 in ATCC43996 indicates that this region was present in isolates before 1995, prior to its acquisition by the new highly virulent strain. VPaI-2 encodes an integrase, a resolvase, hypothetical proteins, a ribonuclease HI, an aminohydrolase, transcriptional regulators and a lipase.

**Table 2:** Primers used in this study

**Primer Designation**	**Primer sequence 5' – 3'**	**Ta (°C)**	**Product size (bp)**
**VPaI-2**			

VP0634F	GGGGGAAATAAATGTCTGAAGG	52.1	1363
VP0634R	AACACGCCAAGACTCTC		
VP0637F	GGAATAACTCAGAGCTTCG	52.8	1848
VP0640R	TAGGCAGTCGTAATTCG		
VP0643F	ATACGCCTGATTGCTTC	52.0	1558
VP0643R	TGGTACTATCAACGCCG		
VP0644F	CGTGCTTTTTCTCTTGC	51.1	970
VP0644R	CCATATTGCTAGTTAGCTCG		

**VPaI-3**			

VP1069F	TAGGGTCGGTGGTGTACTTG	52.8	2041
VP1069R	GACTCCACTATTGGTTTA GC		
VP1072F	AGAGTCAGAGGAAAGGGAGG	50.3	2275
VP1073R	GTAAATGTTGTGGGTGC		
VP1079F	CTGTCTTCATGCCTTTG	51.0	1691
VP1079R	CGCCATTGCTAAACGTC		
VP1083F	CTTACTTATTGGAGGCTGG	52.1	2127
VP1083R	GGTGGGTATAAAGGTAACG		
VP1095F	TCTGGTTCGGTATTTGG	52.7	1166
VP1096R	CGCAGCATTTCTTGAAG		

**VPaI-7**			

VPA1308F	TTAGAACGCATGAGCACCG	53.1	1844
VPA1309R	CCACCAAAGTGTTTGTGAG		
VPA1312F	CTACTATCATCACGACGTG	49.4	1487
VPA1314R	CGTGCTTATAGCCAGAC		
VPA1317F	GACAGACAGAGATACGCTG	50.9	1366
VPA1320R	TTCAGAGGTGTCGCACTTCG		
VPA1321F	CGTGGTGGTTAGTGAATC	49.1	886
VPA1321R	AGAGTTGGTTTCGCAGG		
VPA1321F	GACCACTATATTGTTCTCCG	49.4	1480
VPA1323R	CTCAGGGATAAATAGGGATG		
VPA1331F	CCAATAATCACCCTCCG	49.9	1857
VPA1334R	CTCAGGGATAAATAGGGATG		
VPA1340F	GTCCTTGATTACACCATTGG	51.8	1618
VPA1343R	GCACGTAACATCTAAGTTCGTG		
VPA1350F	TGCATCGTCATTTCTCC	50.4	2630
VPA1354R	CGTAGATTTCATGGCAG		
VPA1363F	TTTCACTAATGCTGCGG	51.1	2308
VPA1365R	GGTCAATATGGCACTATGC		
VPA1380F	TTAGGGGTGTTATGCCG	48.5	816
VPA1380R	TTACTGTCTCTGTTGCAGG		
VPA1390F	CCACAACACAAACTGTCC	50.9	2606
VPA1393R	AATCCAAGGGGAGTGAC		
VPA1394F	AACGCCGAATTAACCGC	53.2	2356
VPA1395R	TCACCCCAATGTACCGTCTG		
VPA1397F	GCGGAGCTGTAATGAAATG	52.9	793
VPA1398R	CAACCAACGTATTGTAGCAG		
VPA1400F	ATAGGTCTGTGTAACCCG	52.3	1964
VPA1401R	GGTAAAGCTGCGATGAC		
VP0085F	TGCTCGCTGCTATCTAC	53.0	1160
VP0085R	CGTTAAATACGCCAGTTGC		
VP0220F	CCCTCAAGTGATTGATCC	53.0	1878
VP0220R	AAGATAGCCCCTTGTGG		
VP1399F	CATCTCTTGCTCTTGGAG	51.0	1312
VP1400R	TGAGGTCTACAATGAGTCAG		
VP1415F	CGCAATTAAAGGCAGTACG	51.0	2274
VP1416R	GACTGAATAAGAGTGCTCG		
VP1556F	TCCCGATTGTAAGTTGC	53.0	1957
VP1558R	AGCTAATGCGAATGAGC		
VPA0446F	ACGTTCTTTTGGGATGG	53.0	2376
VPA0447R	ACCGAAGCCTTAACACG		
VPA0450F	AATGCGAAAGAAGGCGATAC	60.0	1337
VPA0450R	TGCGCTTGTAGATGAGTTGG		
VPA0891F	GTCGCTCTTTATGTTGC	50.5	615
VPA0891R	GAAGCCTGTATCAACTGTC		
VPA0894F	TAATGGTCGATGCACTG	54.0	1215
VPA0894R	GGATGAGCAAGTCAGTAGC		
VPA0952F	CGAGTGATCCAGTTTTACAC	52.0	2720
VPA0953R	AACTACCAGCTAGAAGTGG		
VPA0992F	CCGACATAAAGGGATACTC	52.0	1926
VPA0992R	GAAGAAGCACTTGCTCTC		
VPA1443F	CAATCAGCAGCCAGTCGTTA	60.0	1233
VPA1443R	CCGGATGTCAAACGGTACTT		
VPA1503F	GTTCGACAATGGCATGTGAG	60.0	801
VPA1503R	CGCCAGTATCGACATCACTC		
VPA1655F	CCGTTTTGCTGATGCTACTG	60.0	816
VPA1655R	ATGACCACAGTTCCGGAGAG		
VPGyrBF	GTA CTG AAG GGT CTG GAT GC	54.6	742
VPGyrBR	ACT GCA TTG CCA CTT CTA CC		
VPmdhF	TGAAAGTAGCCGTTATTGG	54.0	901
VPmdhR	CCATTTAGCGTTTCTAGCATTC		
VPGroEL1F	TTTCGGTGCTCCAACCATC	55.4	737
VPGroEL1R	GCATTGCTTTACGACGGTC		

Molecular analysis of the distribution of VPaI-3 found that the region is present in 25 *V. parahaemolyticus *isolates, which included the same set of isolates that contain VPaI-1, VPaI-2, and VPaI-4 to VPaI-6 (O3:K6 and related isolates recovered after 1995) (Fig. 2). One exception was noted, KE10462, an O3:K6 isolate recovered in Japan in 1986. In addition, in two O3:K6 pre-1995 isolates, KE9967 and U-5474, the VPaI-3 region was found to be partially present, suggesting that this region is unstable and has been deleted from these isolates. VPaI-3 contains several transcriptional regulators, hypothetical proteins and a methyl accepting chemotaxis protein.

VPaI-7, an 81 kb region present on chromosome 2, encodes T3SS-2, two copies of the *tdh *gene, a cytotoxic necrotizing factor, an exoenzyme T gene and five transposase genes [20]. T3SS-2 in *V. parahaemolyticus *showed similarity to a T3SS present in several pathogenic *V. cholerae *non-O1 and non-O139 isolates [50,51]. To determine the distribution of VPaI-7, we used 12 primer pairs spanning the 81 kb region (Table 2). Of the 42 *V. parahaemolyticus *isolates examined, 30 isolates were found to contain the entire VPaI-7 region. Similar to the VPaI-2 and VPaI-3 regions, the 30 VPaI-7-positive isolates included all 24 highly virulent isolates as well as 3 O3:K6 strains isolated pre-1995, strains KE9967, U-5474, and ATCC43996 (Fig. 2). The region was present in three O4 serogroup isolates, 1324, an O4:K6, and two Spanish isolates, 30824 and 428/00 (Fig. 2). Although, T3SS-2 was previously reported to be present only in the highly virulent strain, this appears not to be the case as others have found [52]. This region was partially present in 6 isolates (Fig. 2). A primer pair designed within VPA1308/VPA1309, and a primer pair within and VPA1400/VPA1401 all gave a positive PCR product with all strains examined indicating that these genes represent core chromosomal flanking genes.

We examined 11 additional regions that were unique to *V. parahaemolyticus *for their distribution among our collection of isolates, all 11 regions were present in all the highly virulent isolates, in fact 3 regions were present in all strains examined (see Additional file 5). Five regions were absent from 1 to 3 isolates, which included the region that encodes T6SS that is absent from two pre-1995 O3:K6 strains. Three regions were absent from five isolates (see Additional file 5).

### Evolutionary genetic relationships

To understand the evolutionary significance of the distribution of the VPaI regions among our collection of isolates, a phylogenetic frame work was constructed by multilocus sequence (MLS) analysis of an initial analysis of three housekeeping genes. MLS analysis was demonstrated in numerous studies to be a powerful method to both discriminate and determine the phylogenetic relationships among bacterial isolates including *Vibrio *species [53-55]. We found similar to others that *V. parahaemolyticus *isolates are highly related sharing substantial sequence similarity at the three loci we examined [37,42] (Fig. 2). Within the 1854 bps examined among the 42 isolates, there were a total of 39 polymorphic sites of which 21 sites were phylogenetically informative and 12 sequence types (ST) were found. For the purposes of this study, a phylogenetic tree was constructed by the neighbor-joining method using Kimura 2-parameter, which clustered the strains into two closely related but distinct groups named A and B (Fig. 2). The first group contains all highly virulent isolates whereas group B is comprised of mainly environmental isolates recovered in Spain in the early 2000s. Within group A are 25 isolates that are identical at all three loci examined; these isolates include 22 O3:K6 isolates with worldwide distribution recovered pre-1995 and post-1995, and 3 O1 serogroup isolates (O1:K25 and O1:KUT) recovered post-1995. O3:K6 and O1:K25 isolates recovered post-1995, and O3:K6 isolates recovered pre-1995 shared identical sequence profiles (Fig. 2). These data support the hypothesis that O1:K25 and O1:KUT serogroups arose from the O3:K6 highly virulent strain by acquisition of novel O and K antigens similar to the emergence of the pathogenic *V. cholerae *O139 serogroup strain from an O1 El Tor isolate. Acquisition of novel O and K antigens would be evolutionary advantageous since it may play a role in host immune avoidance in *V. parahaemolyticus *infection. Clustering with these O3:K6, O1:K25, O1:KUT isolates are four identical O4 serogroup isolates and KE10464, a divergent O3:K6 pre-1995 (Fig. 2). Thus, it appears that acquisition of novel O antigens is frequent in this species and more recent data suggests this is an ongoing event [3]. Also found within group A are several divergent O3:K6 pre-1995 and post-1995 isolates, and a single divergent O4:K8 post-1995, 1324 (Fig. 2). Group B consists of 5 isolates, with various serotype designations but all were recovered in Spain post-1995 (Fig. 2). Three of the strains were recovered from mollusks and sea sediment, and two strains 30824 and 428/00, which shared an identical ST, were from clinical sources. Overall, the phylogenetic tree constructed from concatenated sequences of three housekeeping genes indicates that pathogenic *V. parahaemolyticus *isolates are highly homologous as others have previously shown [42].

We mapped the distribution of each of the VPaI genomic islands onto the phylogenetic tree to elucidate the possible steps involved in the emergence of the globally distributed *V. parahaemolyticus *new highly virulent strain. We found that similar to VPaI-1, VPaI-4, VPaI-5 and VPaI-6, VPaI-2 and VPaI-3 are predominately present among the highly virulent isolates recovered after 1995 with only one exception noted for VPaI-2, strain ATCC43996 recovered in the UK in 1970, an O3:K4 serogroup (Fig. 2). The VPaI-3 region was present in two pre-1995 O3:K6 isolates, KE9967 and U5474 that have an identical sequence type, and in KE10462, which shows an identical sequence type to five additional pre-1995 and 16 post-1995 O3:K6 isolates. KE10462 also appears to have contained the VPaI-7 regions since it is partially present in this isolate (Fig. 2). KE10462 has been shown to be positive for group specific PCR (GS-PCR), which is based on the *toxRS *nucleotide sequence, that has previously been shown to differentiate post-1995 pandemic strains from non-pandemic and pre-1995 isolates [26,39].

In conclusion, the most parsimonious scenario for the evolution of the new highly virulent O3:K6 clone suggests that a pre-1995 O3:K6 strain obtained regions VPaI-1 to VPI-7, and a T6SS encoded within ORFs VP1386–VP1420, this secretion systems along with T3SS-2 may explain the highly virulent nature of the O3:K6 virulent clone. It appears that *V. parahaemolyticus *isolates have the ability to acquire large regions of DNA and that this is an ongoing process among pathogenic isolates. For example, the O1 and K antigens, which are encoded in the same genomic region, are undergoing frequent change among closely related strains and this may be a mechanism to avoid the host immune system.

The possible origins of the *V. parahaemolyticus *variable regions appear to be quite diverse. Blast analysis of the VPaI-1 encoded proteins found 7 ORFs highly homologous to a 22 Kb island present in *V. cholerae *strain 623–39 at the same tRNA-met insertion site, whereas a similar analysis of VPaI-3 showed high sequence similarity to a region in *V. harveyi *HY01 (AIQ_705 to AIQ_762). VPaI-2 encoded several ORFs with high similarity to ORFs identified in *Vibrio *sp Ex25. Most of VPaI-5 showed homology to ORFs from *Shewanella woodyi *and *Shewanella sp*, and similarly several ORFs of VPaI-6 were homologous to a region in *Shewanella *sp ANA-3. The T3SS-2 region encoded on island VPaI-2 is most closely related to a T3SS recently identified in *V. cholerae *V51, a non-O1 serogroup isolate [56]. Region VP1386 to VP1420, which encodes a T6SS as well as a Rhs element, showed extensive homology to a region in *V. harveyi *ATCC BAA-1116.

## Methods

### Bacterial isolates

A total of 42 *V. parahaemolyticus *isolates were examined in this study as previously described [26]. The 42 isolates were temporally (1970 to 2003) and geographically widespread (Asia, Europe and South America) and encompassed 10 different serotypes. All *V. parahaemolyticus *isolates were grown in Luria-Bertani broth (LB) supplemented with 3% NaCl and stored at -70°C in LB broth with 20% (v/v) glycerol.

### Comparative bioinformatics analysis

We performed four-way BLAST analysis of *V. parahaemolyticus *RIMD2210633, an O3:K6 isolated in 1996, versus *V. vulnificus *YJ016, *V. vulnificus *CMCP6, *V. cholerae *N16961 and *V. fischeri *ES114 to identify regions that were unique to *V. parahaemolyticus*. Complete nucleotide sequences and annotations for the *V. parahaemolyticus *RIMD2210633, *V. vulnificus *YJ016, *V. vulnificus *CMCP6, *V. cholerae *N16961 and *V. fischeri *ES114 were retrieved and downloaded from NCBI [20,57-59]. These were used to construct a genome atlas of the complete genome sequence of all five isolates. The genome atlas plot maps DNA structure features, repeats, and base composition properties of *V. parahaemolyticus *as well as each gene present in RIMD2210633, and their homologues in all four additional species oriented at the *ori*[60]. In addition, we constructed a zoomable genome atlas of the complete genome sequences of all 27 members of the family *Vibrionaceae *available in the database. This data can be interactively examined for chromosome 1 and for chromosome 2 on the web [48]. We compared the genome of *V. parahaemolyticus *RIMD2210633 to the genome of *V. parahaemolyticus *AQ3810, an O3:K6 isolated in 1983, using the Artemis comparison tool (ACT) program [61].

### Molecular analysis

Chromosomal DNA was extracted from each *V. parahaemolyticus *isolate using the G-nome DNA isolation kit from Bio 101. To determine the distribution of regions unique to *V. parahaemolyticus *among our collection of 41 isolates, PCR assays were performed. Primer pairs were designed to target within the regions of interest as well as flanking the regions (Table 2). PCR was performed in a 25 μl reaction mixture with the following cycles: an initial denaturation step at 96°C for 3 min followed by 30 cycles of denaturation at 94°C for 30s, 30s of primer pair annealing at the respective temperature, an extension step at 72°C for 1–4 min (depending on expected PCR product size). PCR primers to amplify three chromosomal housekeeping genes, gyrase subunit B (*gyr*B, VP0014), malate dehydrogenase (*mdh*, VP0325), and chaperonin (*groEL*-1, VP2851), were designed based on the sequence of *V. parahaemolyticus *RIMD2210633 (Table 1). The housekeeping genes were PCR amplified from chromosomal DNA isolated from all *V. parahaemolyticus *isolates and PCR products were purified using Jetquick PCR purification Kit (GENOMED). The *mdh, gyrB *and *groEL-1 *sequences were determined by MWG-Biotech based on the dye deoxy terminator method and the reaction products were separated and detected on an ABI PRISM 3100 genetic analyzer.

### Phylogenetic analysis

The multiple sequence alignment program ClustalW was used to align nucleotide sequences for each housekeeping gene [62]. Rates of synonymous substitutions/synonymous site (K_*S*_) were calculated by the methods of Nei and Gojobori and Nei and Lin [63,64]. To analyze the evolutionary relationships among *V. parahaemolyticus *isolates, the concatenated sequence of all three housekeeping genes was used to construct a Neighbour-Joining phylogenetic tree based on Kimura 2-parameter distance using MEGA-3 [65].

### Nucleotide sequence accession no

The sequences of *mdh, gyrB *and *groEL-1 *were submitted to GenBank and given the accession numbers GenBank EU629305–EU629345.

## Abbreviations

T3SS: type III secretion systems; T6SS: type VI secretion system; VPaI: *Vibrio parahaemolyticus *genomic islands; TDH: thermostable direct hemolysin; KP: Kanagawa-phenomenon; TRH: TDH-related hemolysin; LPS: lipopolysaccaride; CPS: capsule polysaccharide (CPS).

## Authors' contributions

ALC and LMN performed bacteriological, genetic and phylogenetic studies, helped with the experimental design and drafted the manuscript, OCS was involved in the genome sequencing, annotation and drafted the manuscript, TTB and DWU performed genome BLAST atlas analysis. MAP was involved in the experimental design and drafted the manuscript. All authors read and approved the final manuscript.

## Supplementary Material

Additional file 1 **Fig. S1.**  Genome BLAST Atlas of *V. parahaemolyticus* RIMD2210633 as reference strain (inner most circle) versus 27 genomes of members of the family *Vibrionaceae* for chromosome 1. *V. parahaemolyticus* RIMD2210633 as reference strain (inner most circle) versus *V. parahaemolyticus* AQ3810, *V. cholerae* 1587, AM-19226, MAK757, MO10, MZO-2, MZO-3, B33, NCTC8457, RC385, O395, V51, V52, 623-39, and 2740-80, *V. harveyi* ATCCBAA116, *V. alginolyticus* 12G01, *Vibrio* sp. Ex25, *V. vulnificus* CMCP6 and YJ016, *V. splendidus* 12B01, *Vibrio* sp. MED222, *V. fischeri* ES114, *V. salmonicida* LF1238, *V. angustum* S14, *P. profundum* SS9 and 3TCK for chromosome 1.  The gaps or holes in the outer four circles represent regions present in *V. parahaemolyticus* strain RIMD2210633 that are absent from the other species.  The innermost circles show DNA structure features, DNA stacking energy, DNA position preference, positive and negative coding strands are indicated by dark blue and red circle. Global direct and global inverted repeats are represented and the two inner most circles represent GC shew and AT content, respectively. Click here for file

Additional file 2 **Fig. S2.**  Genome BLAST Atlas of *V. parahaemolyticus* RIMD2210633 as reference strain (inner most circle) versus 27 genomes of members of the family *Vibrionaceae* for chromosome 2. *V. parahaemolyticus* RIMD2210633 as reference strain (inner most circle) versus *V. parahaemolyticus* AQ3810, *V. cholerae* 1587, AM-19226, MAK757, MO10, MZO-2, MZO-3, B33, NCTC8457, RC385, O395, V51, V52, 623-39, and 2740-80, *V. harveyi* ATCCBAA116, *V. alginolyticus* 12G01, *Vibrio* sp. Ex25, *V. vulnificus* CMCP6 and YJ016, *V. splendidus* 12B01, *Vibrio* sp. MED222, *V. fischeri* ES114, *V. salmonicida* LF1238, *V. angustum* S14, *P. profundum* SS9 and 3TCK for chromosome 2.  The gaps or holes in the outer four circles represent regions present in *V. parahaemolyticus* strain RIMD2210633 that are absent from the other species.  The innermost circles show DNA structure features, DNA stacking energy, DNA position preference, positive and negative coding strands are indicated by dark blue and red circle. Global direct and global inverted repeats are represented and the two inner most circles represent GC shew and AT content, respectively. Click here for file

Additional file 3 **Fig. S3.**  Linear comparison of *V. parahaemolyticus* RIMD2210633 and AQ3810 created using ACT (Artemis Comparison Tool) at the insertion sites of (A) VPaI-1 and VPaI-4, and (B) VPaI-5 and VPaI-6.  A homologous block of genomic sequence (BLASTN matches) is indicated by red lines between the chromosomal regions examined.  The location of the genomic islands (GIs) identified in RIMD2210633 are illustrated above, and in AQ3810 below the genome comparison.  Horizontal arrows represent annotated genes, striped arrows represent integrases, and the direction of the arrow indicates gene orientation. Click here for file

Additional file 4 **Fig. S4.**  Linear comparison of *V. parahaemolyticus* strain RIMD2210633 and strain AQ3810 created using ACT [59] at the insertion sites of (A) VPaI-2 and VPaI-3, and (B) VPaI-9 and  VPaI-10.  A homologous block of genomic sequence (BLASTN matches) is indicated by red and blue lines between the chromosomes; blue lines indicate chromosomal inversion events.  The location of *Vibrio parahaemolyticus* islands (VPaIs) identified is illustrated above for RIMD2210633 and below for AQ3810 the region examined. Horizontal arrows represent annotated genes, striped arrows represent integrases, and the direction of the arrow indicates gene orientation. Click here for file

Additional file 5 **Table S1.** PCR assays of the distribution of 11 regions unique to *V. parahaemolyticus*. Click here for file
