# Supplementary figures and images for: Molecular analysis of the emergence of pandemic Vibrio parahaemolyticus
Source: BMC Microbiol. 2008 Jun 30;8:110. doi: 10.1186/1471-2180-8-110 (PMC2491623; doi:10.1186/1471-2180-8-110)

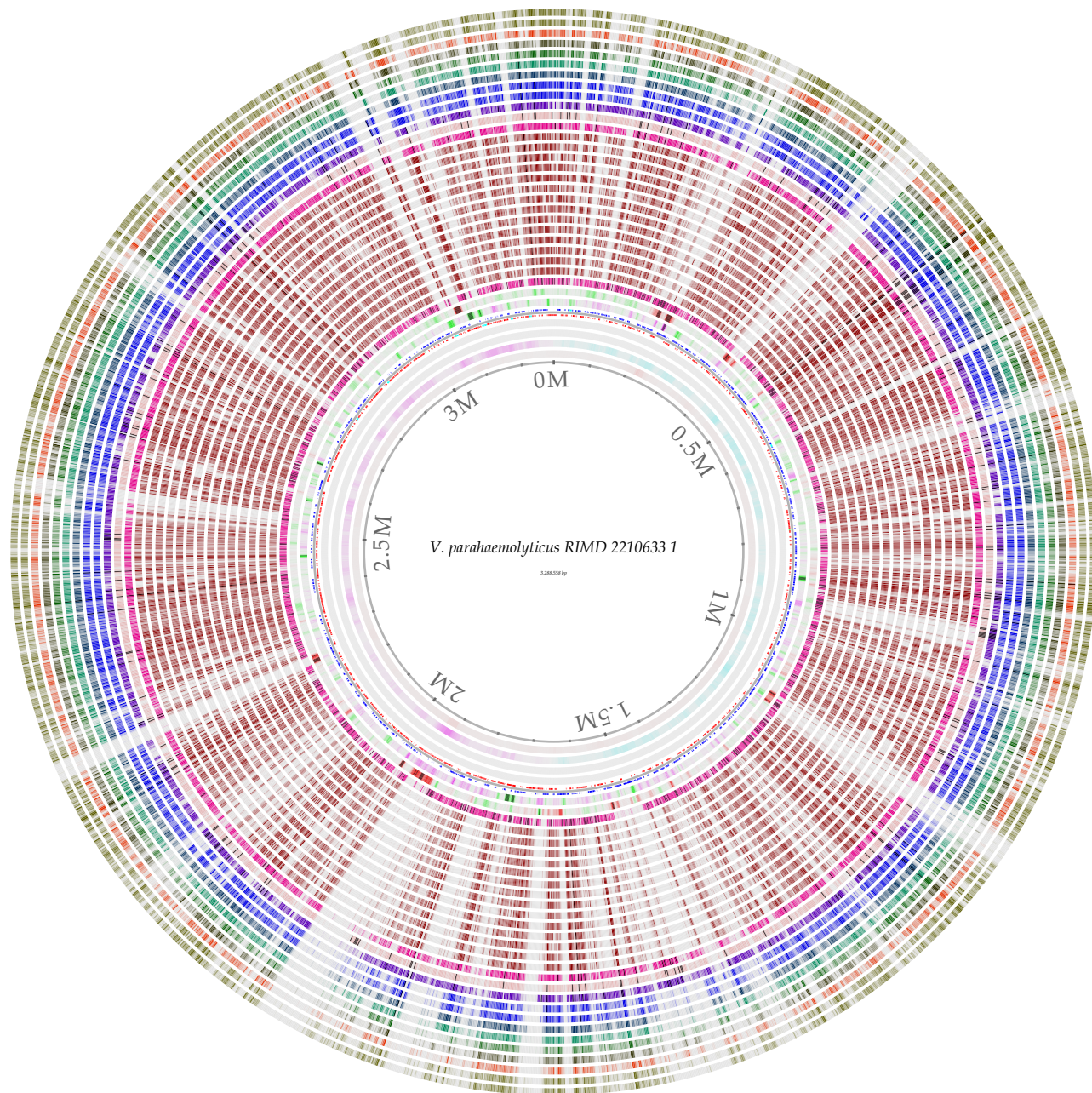

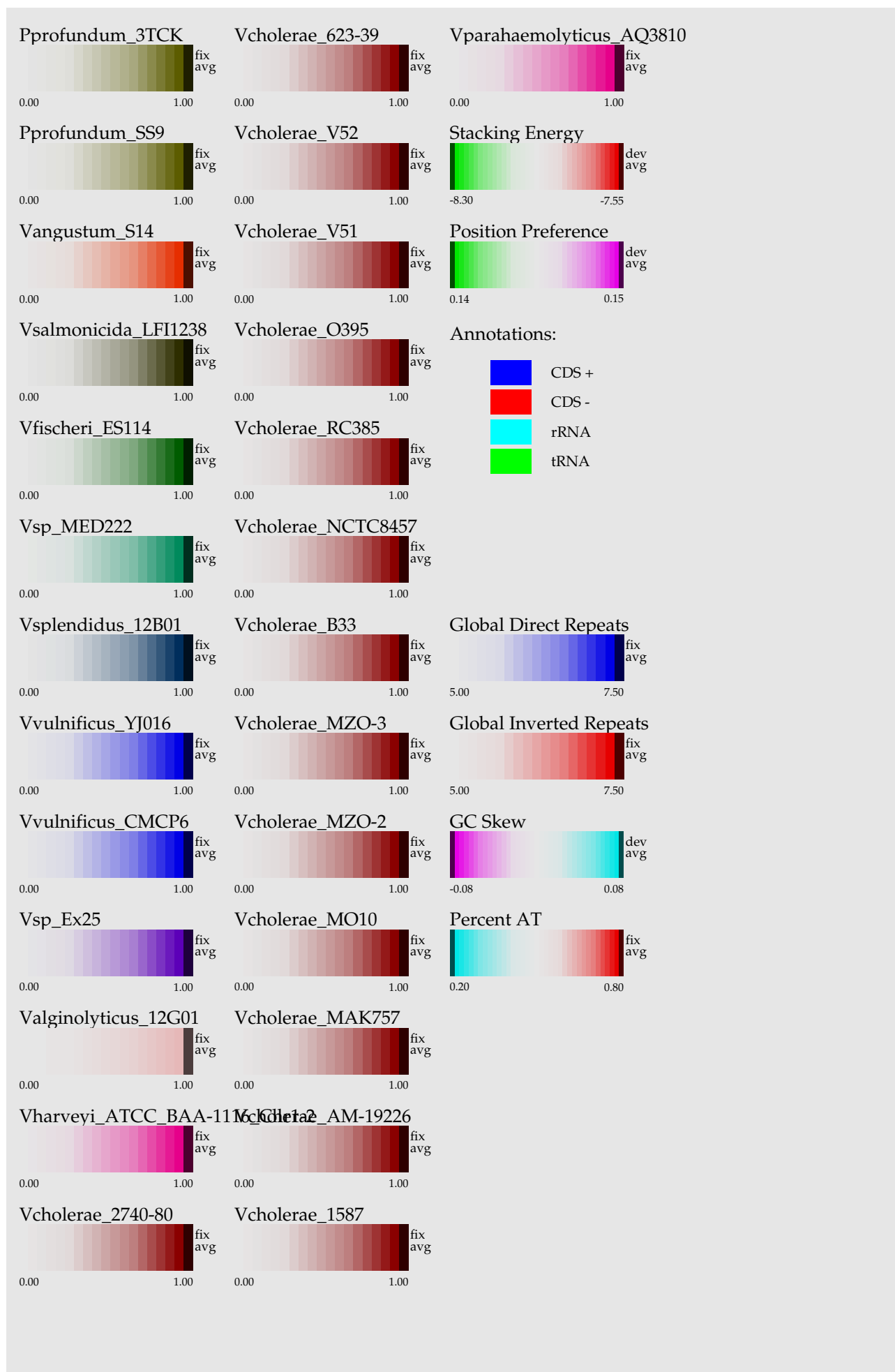

Supplement: Additional file 1 — Fig. S1. Genome BLAST Atlas of V. parahaemolyticus RIMD2210633 as reference strain (inner most circle) versus 27 genomes of members of the family Vibrionaceae for chromosome 1. V. parahaemolyticus RIMD2210633 as reference strain (inner most circle) versus V. parahaemolyticus AQ3810, V. cholerae 1587, AM-19226, MAK757, MO10, MZO-2, MZO-3, B33, NCTC8457, RC385, O395, V51, V52, 623-39, and 2740-80, V. harveyi ATCCBAA116, V. alginolyticus 12G01, Vibrio sp. Ex25, V. vulnificus CMCP6 and YJ016, V. splendidus 12B01, Vibrio sp. MED222, V. fischeri ES114, V. salmonicida LF1238, V. angustum S14, P. profundum SS9 and 3TCK for chromosome 1. The gaps or holes in the outer four circles represent regions present in V. parahaemolyticus strain RIMD2210633 that are absent from the other species. The innermost circles show DNA structure features, DNA stacking energy, DNA position preference, positive and negative coding strands are indicated by dark blue and red circle. Global direct and global inverted repeats are represented and the two inner most circles represent GC shew and AT content, respectively. [file 1471-2180-8-110-S1.pdf]

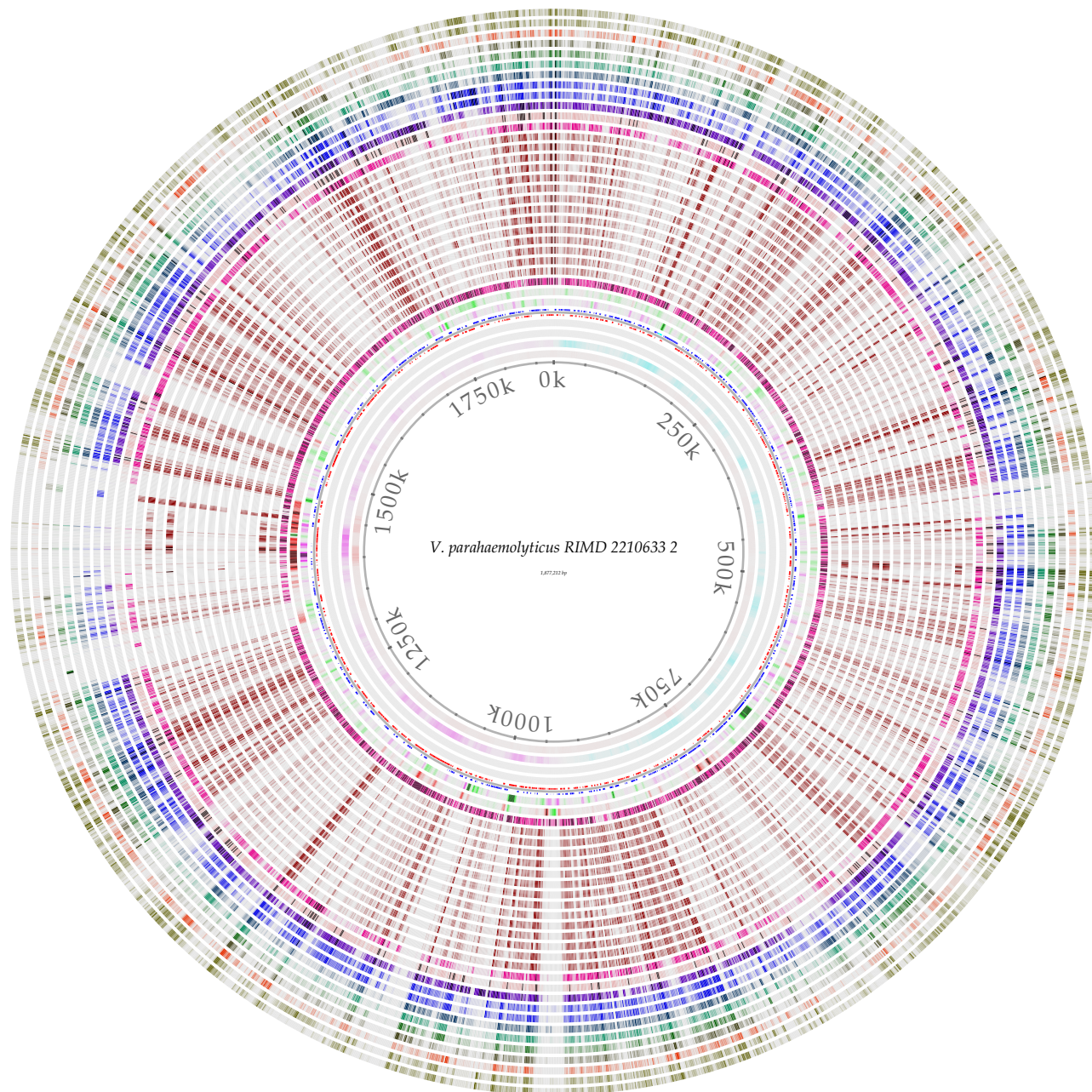

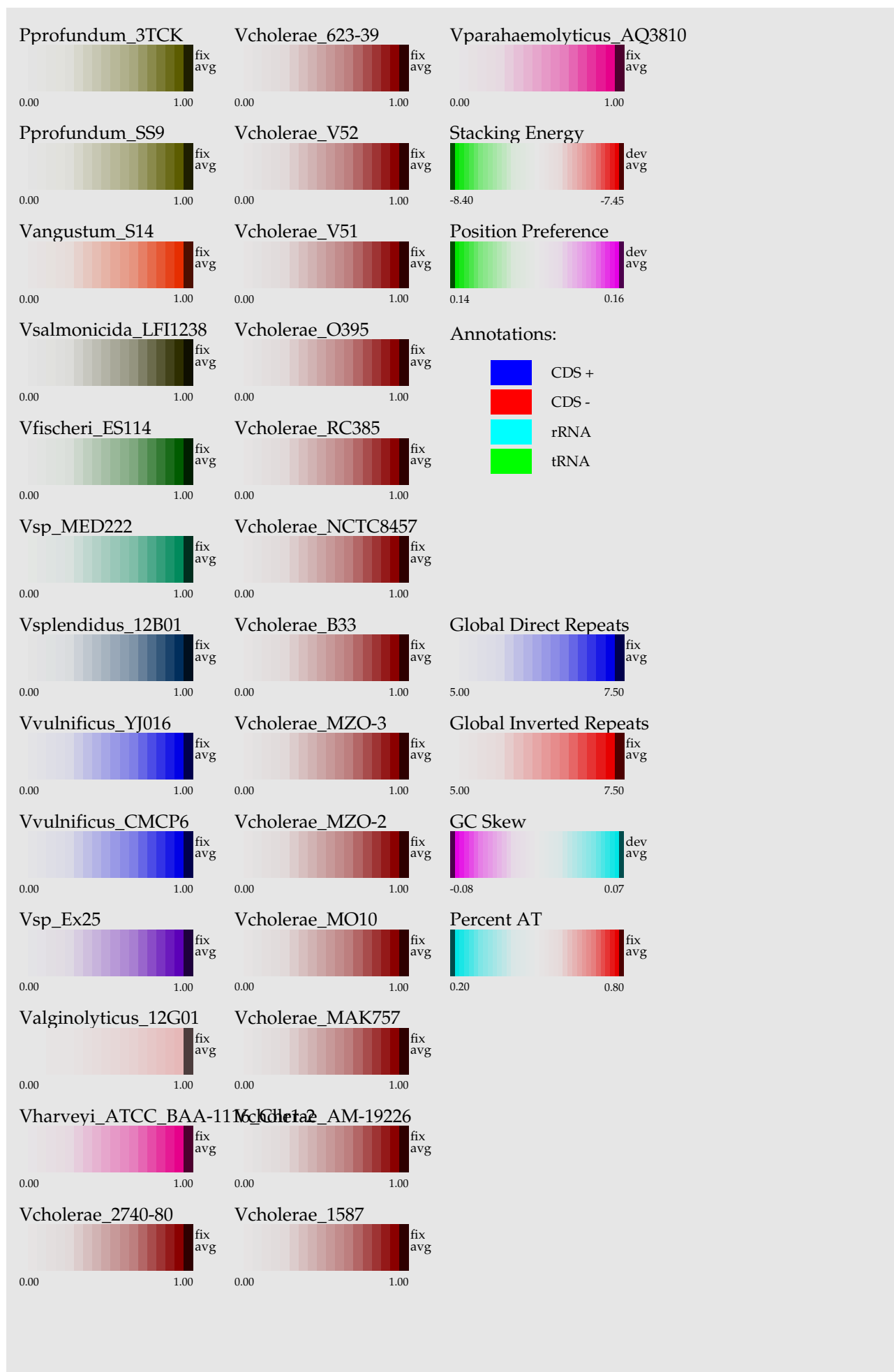

Supplement: Additional file 2 — Fig. S2. Genome BLAST Atlas of V. parahaemolyticus RIMD2210633 as reference strain (inner most circle) versus 27 genomes of members of the family Vibrionaceae for chromosome 2. V. parahaemolyticus RIMD2210633 as reference strain (inner most circle) versus V. parahaemolyticus AQ3810, V. cholerae 1587, AM-19226, MAK757, MO10, MZO-2, MZO-3, B33, NCTC8457, RC385, O395, V51, V52, 623-39, and 2740-80, V. harveyi ATCCBAA116, V. alginolyticus 12G01, Vibrio sp. Ex25, V. vulnificus CMCP6 and YJ016, V. splendidus 12B01, Vibrio sp. MED222, V. fischeri ES114, V. salmonicida LF1238, V. angustum S14, P. profundum SS9 and 3TCK for chromosome 2. The gaps or holes in the outer four circles represent regions present in V. parahaemolyticus strain RIMD2210633 that are absent from the other species. The innermost circles show DNA structure features, DNA stacking energy, DNA position preference, positive and negative coding strands are indicated by dark blue and red circle. Global direct and global inverted repeats are represented and the two inner most circles represent GC shew and AT content, respectively. [file 1471-2180-8-110-S2.pdf]
