# Supplementary material for: Molecular analysis of the emergence of pandemic Vibrio parahaemolyticus
Source: BMC Microbiol. 2008 Jun 30;8:110. doi: 10.1186/1471-2180-8-110 (PMC2491623; doi:10.1186/1471-2180-8-110)
Supplement: Additional file 3 — Fig. S3. Linear comparison of V. parahaemolyticus RIMD2210633 and AQ3810 created using ACT (Artemis Comparison Tool) at the insertion sites of (A) VPaI-1 and VPaI-4, and (B) VPaI-5 and VPaI-6. A homologous block of genomic sequence (BLASTN matches) is indicated by red lines between the chromosomal regions examined. The location of the genomic islands (GIs) identified in RIMD2210633 are illustrated above, and in AQ3810 below the genome comparison. Horizontal arrows represent annotated genes, striped arrows represent integrases, and the direction of the arrow indicates gene orientation. [file 1471-2180-8-110-S3.ppt]

## Slide 1
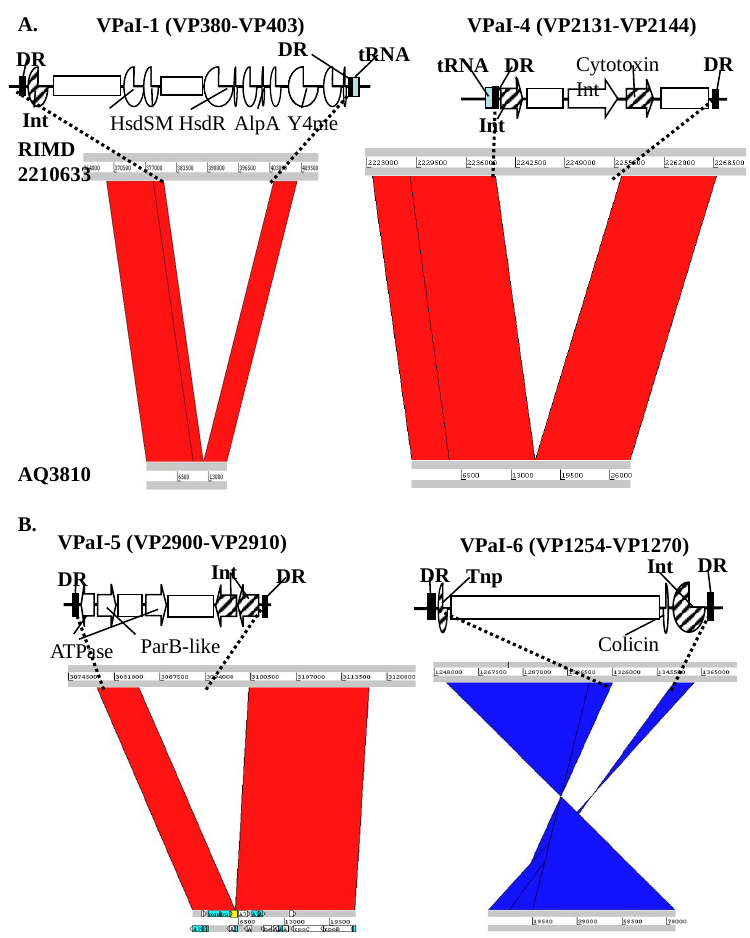

A.
RIMD
2210633
AQ3810
B.
VPaI-1 (VP380-VP403)
VPaI-4 (VP2131-VP2144)
DR
tRNA
DR
Cytotoxin Int
DR
tRNA
DR
Int
AlpA
 HsdSM HsdR
Y4me
Int
VPaI-5 (VP2900-VP2910)
VPaI-6 (VP1254-VP1270)
DR
Int
Int
DR
Tnp
DR
DR
Colicin
ParB-like
ATPase
