# Supplementary material for: Molecular analysis of the emergence of pandemic Vibrio parahaemolyticus
Source: BMC Microbiol. 2008 Jun 30;8:110. doi: 10.1186/1471-2180-8-110 (PMC2491623; doi:10.1186/1471-2180-8-110)
Supplement: Additional file 4 — Fig. S4. Linear comparison of V. parahaemolyticus strain RIMD2210633 and strain AQ3810 created using ACT [59] at the insertion sites of (A) VPaI-2 and VPaI-3, and (B) VPaI-9 and VPaI-10. A homologous block of genomic sequence (BLASTN matches) is indicated by red and blue lines between the chromosomes; blue lines indicate chromosomal inversion events. The location of Vibrio parahaemolyticus islands (VPaIs) identified is illustrated above for RIMD2210633 and below for AQ3810 the region examined. Horizontal arrows represent annotated genes, striped arrows represent integrases, and the direction of the arrow indicates gene orientation. [file 1471-2180-8-110-S4.ppt]

## Slide 1
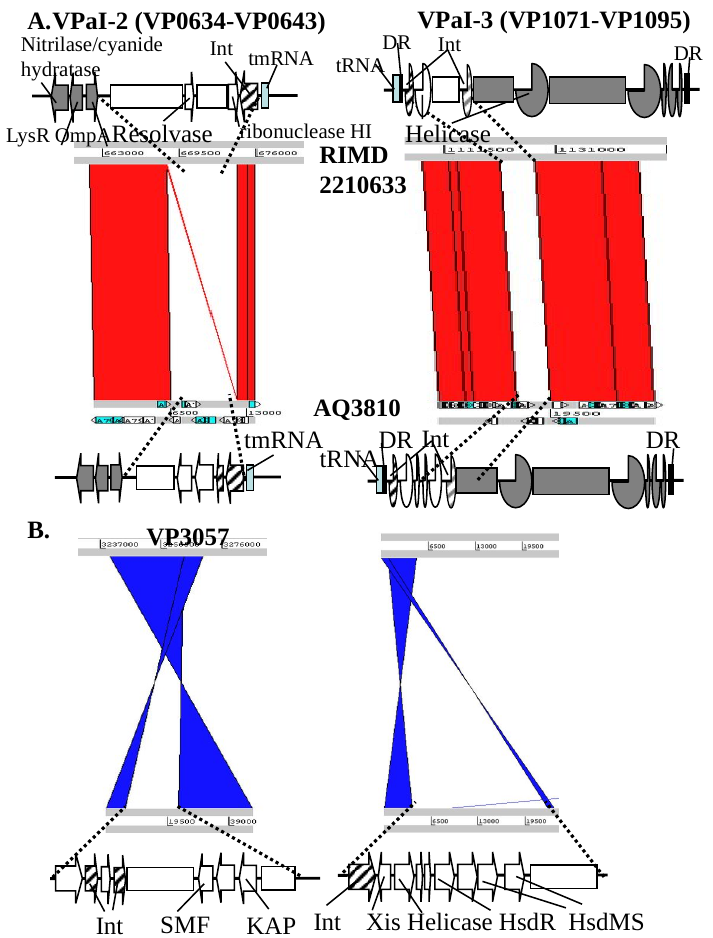

VPaI-3 (VP1071-VP1095)
VPaI-2 (VP0634-VP0643)
A.
B.
DR
Int
DR
tRNA
Nitrilase/cyanide
hydratase
Int
tmRNA
Helicase
ribonuclease HI
Resolvase
LysR OmpA
RIMD
2210633
AQ3810
Int
DR
tmRNA
DR
tRNA
VP3057
Int Xis Helicase HsdR HsdMS
SMF
Int
KAP
