# Supplementary material for: Molecular analysis of the emergence of pandemic Vibrio parahaemolyticus
Source: BMC Microbiol. 2008 Jun 30;8:110. doi: 10.1186/1471-2180-8-110 (PMC2491623; doi:10.1186/1471-2180-8-110)
Supplement: Additional file 5 — Table S1. PCR assays of the distribution of 11 regions unique to V. parahaemolyticus. [file 1471-2180-8-110-S5.doc]

**TABLE S1**. PCR assays of the distribution of 11 regions unique to *V. parahaemolyticus*
